# s-CAM: An Untethered Insertable Laparoscopic Surgical Camera Robot with Non-Contact Actuation

**DOI:** 10.3390/s22093405

**Published:** 2022-04-29

**Authors:** Ning Li, Hui Liu, Reza Yazdanpanah Abdolmalaki, Gregory J. Mancini, Jindong Tan

**Affiliations:** 1 Department of Mechanical, Aerospace and Biomedical Engineering, University of Tennessee, Knoxville, TN 37996, USA or lining@azrmed.com (N.L.); hliu58@vols.utk.edu (H.L.); ryazdanp@vols.utk.edu (R.Y.A.); 2 Azure Medical Innovation Corporation, Knoxville, TN 37922, USA; 3 University of Tennessee Medical Center, Knoxville, TN 37920, USA; gmancini@utmck.edu

**Keywords:** insertable laparoscopic camera, robotic-assisted surgery, minimally invasive surgery, medical robotics

## Abstract

Fully insertable robotic imaging devices represent a promising future of minimally invasive laparoscopic vision. Emerging research efforts in this field have resulted in several proof-of-concept prototypes. One common drawback of these designs derives from their clumsy tethering wires which not only cause operational interference but also reduce camera mobility. In this paper, a tetherless insertable surgical camera (s-CAM) robot with non-contact transabdominal actuation is presented for single-incision laparoscopic vision. Wireless video transmission and control communication using onboard power help eliminate cumbersome tethering wires. Furthermore, magnetic based camera actuation gets rid of intrinsic physical constraints of mechanical driving mechanisms, thereby improving camera mobility and reducing operational interference. In addition, a custom Bluetooth low energy (BLE) application profile and a real-time operating system (RTOS) based multitask programming framework are also proposed to facilitate embedded software design for insertable medical devices. Initial ex vivo test results of the s-CAM design have demonstrated technical feasibility of a tetherless insertable laparoscopic camera. Effective imaging is confirmed at as low as 500 lx illumination. Wireless laparoscopic vision is accessible within a distance of more than 10 m. Transabdominal BLE communication is stable at over −52 dBm and shows its potential for wireless control of insertable medical devices. RTOS based sfotware event response is bounded within 1 ms while the CPU usage is at 3∼5%. The device is able to work for 50 min with its onboard power. For the mobility, the robot can translate against the interior abdominal wall to reach full abdomen quadrants, tilt between −180${}^{\circ }$ and +180${}^{\circ }$, and pan in the range of 0${}^{\circ }$∼360${}^{\circ }$. The s-CAM has brought robotic laparoscopic imaging one step further toward less invasiveness and more dexterity.

## 1. Introduction

Aiming to reduce the number of incisions and thereby make laparoscopic surgery (LS) less invasive, single-incision laparoscopic surgery (SILS) has been introduced and begun to prevail. Compared to traditional multi-port laparoscopic surgery (MLS), SILS has benefited patients with lower level of discomfort, less postoperative pain, faster recovery, as well as better scarring results [1]. However, one noteworthy problem is that the ease of operation for surgeons has also been hindered by loss of triangulation [2] and increased clashing between surgical devices at the shared entry port. To meet these operational challenges, researchers and engineers have been working towards novel surgical devices and platforms [3,4,5,6,7,8,9,10] with improved ergonomics and flexibility. One of the most common piece of equipment required for SILS is the the laparoscope [11], which is an essential telescopic imaging device that allows viewing the internal surgical site from the outside. This device plays an irreplaceable role in surgical imaging, however it makes the shared SILS port more crowded and suffers from kinematic limitations due to the inherited trocar-based paradigm. A paradigm-shifting laparoscopic imaging technique breaking current limitations will effectively advance SILS instrumentation and boost medical science progress.

**Figure 1:**
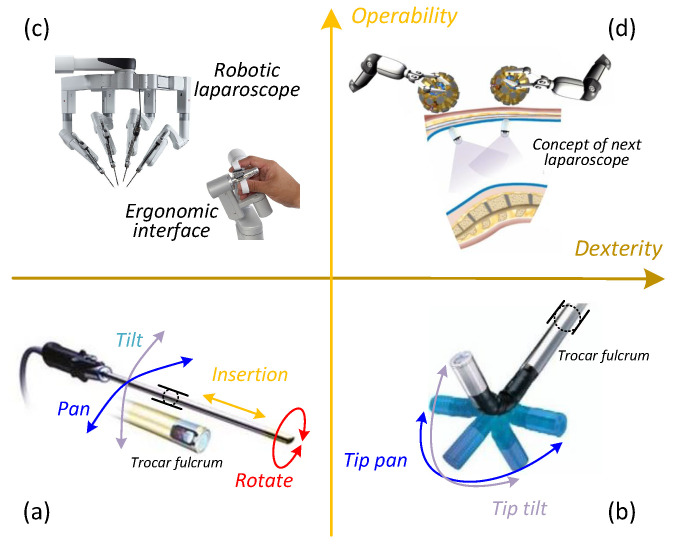
Laparoscope paradigm evolution in terms of operability and dexterity. (**a**) Traditional laparoscope confined by trocar, (**b**) Laparoscope with an articulating tip, (**c**) Robotic-assisted laparoscope, (**d**) Future laparoscope with dexterous mobility and intuitive operability.

Evolution of laparoscope paradigms can be interpreted in terms of their dexterity and operability as indicated in Figure 1. Thanks to advances in biomechatronics, delicate mechanisms and robotic features are being incorporated to improve the state of the art. The most conventional laparoscope [11] shown in Figure 1a is a rigid slender endoscope with a trocar-confined 4-DoF workspace [12]. In order to obtain a larger field of observation, an articulating tip was introduced [13,14] as indicated in Figure 1b. This design added two degrees of articulation and subsequently enabled full abdominal observation, although sometimes at an inferior angle of view. However, suffering from the trocar fulcrum effect, these designs significantly depend on counter-intuitive manual control and hand-eye coordination of a well-trained laparoscopist. That is why robotics has found its way into the operating room (OR) [15,16] with the potential to improve surgical operability and remove the steep learning curve.

Best manifested by the *da Vinci^®^* surgical systems (Xi, Single-Site, and SP) represented by Figure 1c, robotic-assisted laparoscopes have provided an intuitive surgeon interface with unprecedented ergonomics and precision. Unfortunately, due to inherent trocar channel constraints, movement of the long rigid laparoscope is still confined to the same limited workspace as before. Occasionally, a second cut becomes inevitable for laparoscope replacement to get a preferred view angle, which may convert a SILS surgery to a MLS or even an open surgery [17,18]. In summary, the current clinical state of laparoscopes necessitates incisions, accounts for instrument clashing, and therefore is becoming a bottleneck of modern medical progress. Following the evolution trending displayed in Figure 1, the next desirable generation of laparoscope should appear as Figure 1d and be characterized by dexterous mobility and intuitive operability.

Recent efforts in shaping the next generation of laparoscope are being driven by the idea of laparoscopic camera which can be fully inserted and operated remotely inside the human abdominal cavity. Starting from mechanical anchoring, motorized actuation, and wired tethering, related work have been advancing in two directions: non-contact actuation and tetherless access. Hu et al. introduced a cable-tethered insertable surgical imaging device with various implementations [7,19]. By contrast, MARVEL [8,20] designed by Castro et al. is a cable-free motorized robotic pan/tilt surgical camera. Obviously, both suturing and piercing fixate the camera mechanically and cause extra invasiveness, making repositioning of the camera difficult if not impossible. Instead, a magnetic levitated laparoscopic imaging robot designed by Simi et al. [9,21] showed the ability to translate the camera by magnetic coupling. Recognizing operational interference caused by tethering wires from their previous work [10,22], Platt et al. presented a wireless design of a wheeled ceiling pan/tilt robot with magnetic anchoring and motorized mobility [23]. Another two platforms with magnetic anchoring and motorized actuation were contributed, respectively, by Menciassi et al. [5,24] and Fu et al. [25]. Magnetic coupling enables improved mobility of insertable laparoscopic devices, while the onboard motors and actuation mechanisms keep these prototypes mechanically bulky and complicated. Pure magnetic actuation for an insertable laparoscopic camera was presented by Garbini et al. [26,27]. A small form factor has been achieved by eliminating motors and complicated actuation mechanisms except that a bundle of tethering wires were still required. However, operational interference caused by tethering wires has been recognized as a common drawback of current prototypes [23,28]. As is reported by studies [23,29,30], increasing the number of wires in the tether reduces its overall flexibility and thus affects mobility of the tethered camera. So far, exploring steps have been taken towards eliminating mechanical mechanisms and physical tethering for insertable laparoscopic cameras. Unfortunately, each solution was only able to partially meet the non-contact actuation and tetherless control expectations.

The goal of this paper is to address the drawbacks with state-of-the-art designs related to their cumbersome tethering wires and mechanical locomotion constraints. Our previous work resulted in an actuation mechanism based on a combination of permanent magnets and electromagnets [31], where a dummy camera was being used for open-loop actuation evaluation. In this paper, we have been able to contribute a fully functional tetherless insertable laparoscopic surgical camera (s-CAM) with pure permanent magnetic actuation and improved camera mobility. This work also shares a modular hardware architecture, a proprietary BLE application profile, and a reliable and safety-critical software framework to promote development of general insertable surgical robots.

The objectives of this s-CAM system include (i) realizing non-contact camera actuation across the abdominal wall, (ii) achieving wireless laparoscopic vision and control communication, while (iii) guaranteeing medical compatibility, safety, and reliability. However, non-contact to access from the outside, this camera needs to leverage cutting-edge biomechatronics under stringent limitations to establish wireless links and surgical sensing based on limited onboard resources. Unique technological and engineering challenges arise from physical and medical restrictions for design, implementation, and clinical acceptance of this fully insertable laparoscopic camera. Mechanically, a small form factor and a light weight are always preferred for this minimally invasive surgical camera. Therefore, electrically, outfitting this camera robot features integrating just right payloads within a compact profile. Clinically, medical safety and reliability should be emphasized according to application requirements. Meanwhile, this camera must be compatible with existing MIS tools so that it can be successfully introduced and properly fitted into the OR.

The remaining of this article is organized as follows: Section 2 gives an overview of the s-CAM concept, relevant clinical protocol, as well as its design objectives and challenges; Section 3 provides a detailed description of non-contact actuation and mobility of the insertable camera; while implementation of tetherless vision and control is elaborated on in Section 4; and embedded software development is interpreted in Section 5; experimental results presented in Section 6 demonstrate basic s-CAM functions and validate the design feasibility; finally, some concluding remarks and vision into future are shared in Section 7.

## 2. s-CAM Concept and Principle

**Figure 2:**
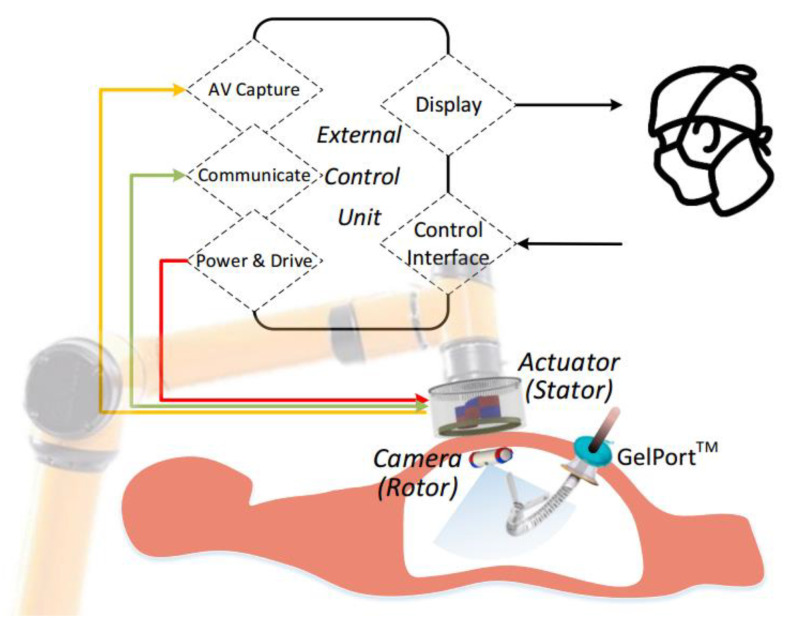
s-CAM concept and working principle. An AUBO-i5™ [32] collaborative robotic arm (Smokie Robotics, Inc., Knoxville, TN, USA), a continuum robotic manipulator (Titan Medical Inc., Toronto, ON, Canada) and a GelPort^®^ SILS access port (Applied Medical Resources Corporation, Rancho Santa Margarita, CA, USA) are included for technical reference.

Concept and working principle of the s-CAM is illustrated in Figure 2, which features a self-contained robotic laparoscopic imaging system that may either work independently in a diagnostic procedure or function as part of an integral robotic surgical system. The s-CAM system consists of an insertable laparoscopic camera, an actuator held by a collaborative robotic arm, and an external control unit (ECU). Magnetic-based transabdominal camera actuation eliminates mechanical fixations or mechanisms, and thus lays foundation for flexible camera mobility. Laparoscopic audio/video (AV) and control communications between the camera and the actuator are realized in wireless manners, which helps remove cumbersome tethering wires.

A lightweight collaborative robotic arm capable of force sensing and collision detection is adopted for manipulating the actuator, which not only provides robotic assistance but also interacts safely with surgeons. This robotic arm stops in milliseconds once an overload or collision event is detected. Different from teleoperated slave robots, this robot arm collaborates and interacts with surgeons side by side. By manually dragging the collaborative arm, surgeons may take control of the actuator intraoperatively in case of an incident and reposition it arbitrarily to a desired safe position to avoid secondary harms. The actuator is reusable after appropriate sterilization as it may be covered with a drape during application while the camera is disposable.

The ECU is the control center of the s-CAM system, serving as a bridge between the surgeon and the robotic camera. On the robotic camera side, the ECU controls the robotic arm to manipulate the actuator which finally drives the camera. At the same time, the ECU accesses AV signals from, communicates with, and controls the power of the actuator. On the surgeon’s side, the ECU provides an ergonomic user interface for intuitive camera manipulation with real-time video display. In this manner, behavior of the in vivo camera is precisely controlled from a remote console.

## 3. Non-Contact Camera Actuation

**Figure 3:**
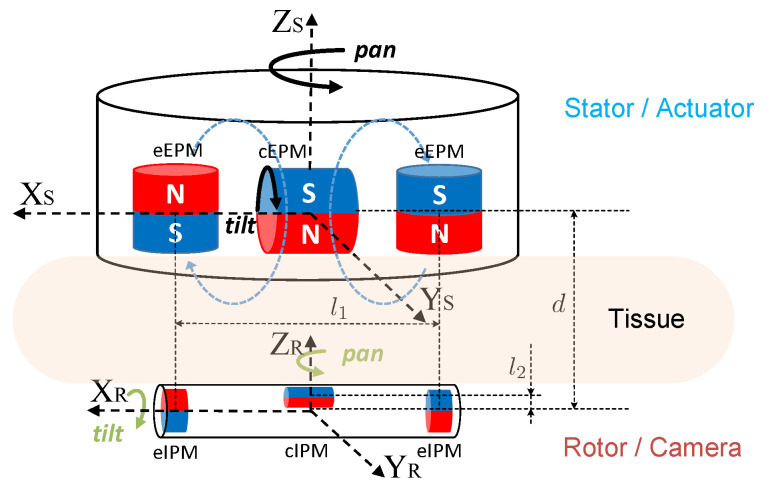
Magnetic-based stator-rotor actuation mechanism.

Inspired by the principle of spherical motors which enables multi-axis rotor rotation with one compact magnetic rolling joint [33,34], actuation of this camera is realized in a variant non-contact stator-rotor manner as is schematically illustrated in Figure 3. $X_{S}Y_{S}Z_{S}$ is the stator coordinate frame and $X_{R}Y_{R}Z_{R}$ is the rotor coordinate frame. Permanent magnets on the stator are operated by incorporated motors to generate controllable rotating magnetic fields. Thus, adjustable tightly coupled magnetic attraction between the stator (actuator) and the rotor (camera) provides manipulating forces and torques for driving this in vivo camera. Magnetic dipole model based on Maxwell equations was used to formulate analytical solutions of actuation force and torque on the camera [31]. Meanwhile, finite element analysis (FEA) based numerical solution was simulated in COMSOL using mash differentiation in spatial domain due to the quasi-static feature of the system [35]. The analytical and numerical solutions were verified using a 6-D force/torque sensor [31,36]. Pan, tilt, translation as well as anchoring of the camera are all enabled by actively generating desired driving magnetic fields in accordance to the camera pose.

### 3.1. Decoupled Camera Mobility: Anchoring, Translation, Pan and Tilt

In order to meet SILS imaging requirement of observing the whole abdominal cavity from any desired position and angle over the surgical site, 4 appropriate DoFs in camera mobility will be sufficient. Although traditional rigid laparoscopes also have 4 or even more DoFs, they are confined by the trocar channel and can only pivot around the fulcrum at the entry port, which prevents arbitrarily orienting the tip [12]. For the s-CAM, 2-DoF translation along the abdominal wall moves the camera to any desired position while 2-DoF rotation directs the camera in any desired perspective. These mobility DoFs are decoupled by design to simplify kinematic modeling and control of the camera. Anchoring of the camera is supported by magnetic attraction forces between all external permanent magnets (EPMs) and internal permanent magnets (IPMs). Appropriate camera–tissue interaction force may be maintained by adjusting the distance betweent the actuator and the camera [36]. Facilitated by these attraction forces, the camera can be translated along $X_{R}$ and $Y_{R}$ axes by moving the stator in corresponding directions. It is worth noting that an appropriate camera–tissue contact force [37,38] should be maintained so that the camera will neither fall off nor damage tissues [36,39]. As is shown in Figure 3, the camera pans about $Z_{R}$ axis enabled by the rotation of the stator which carries all EPMs. Meanwhile, tilt motion of the camera about $X_{R}$ axis is achieved by magnetic coupling between the central internal permanent magnet (cIPM) and the central external permanent magnet (cEPM). Details of magnets used on this robot are provided in Table 1. In this way, rotational motion of the camera is decoupled and orientation control is simplified. As a result, this s-CAM system enables multi-quadrant omnidirectional laparoscopic imaging with 4 decoupled DoFs.

### 3.2. Rotor Design and Fabrication

**Figure 4:**
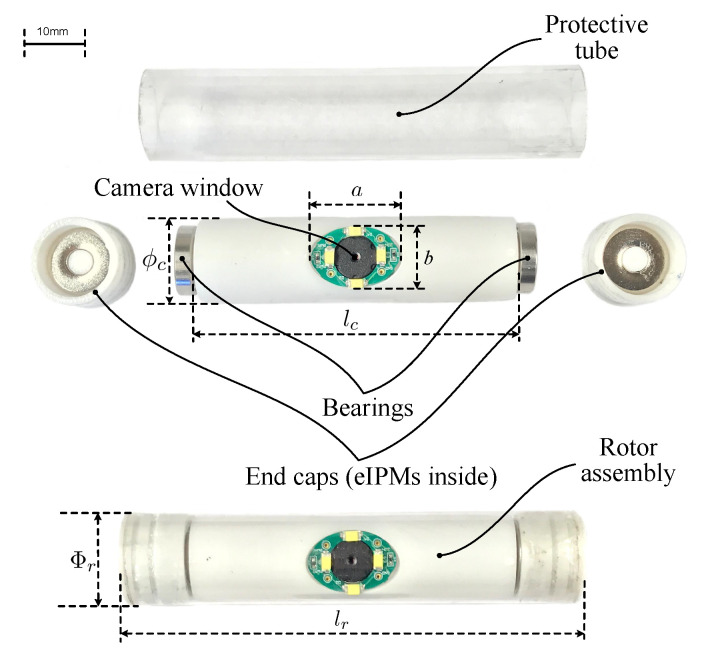
Mechanical design and fabrication of the rotor.

The s-CAM traverses across the abdominal cavity against the interior abdominal wall and has been designed as the rotor. Figure 3 and Figure 4, respectively, present its magnetic schematic and mechanical implementation. Three diametrically magnetized IPMs are installed for actuation purpose. One cIPM is attached inside the camera body and moves with the camera as one rigid piece. Meanwhile, two end internal permanent magnets (eIPMs) are fitted inside two end caps which are mounted at the camera ends through two bearings, as is shown in the implementation (Figure 4). In this way, the camera is able to tilt with respect to the eIPMs. Moreover, an oval window is opened sideways in the middle for camera view and illumination. Housing of the camera is printed with biocompatible resin using a Formlabs Form 2^®^ 3D printer. Finally, the whole camera assembly has been encapsuled into a biocompatible transparent polyvinylchloride (PVC) tube, which temporarily prevents the lens from getting blurred. The finished rotor profile resembles a cylinder of ϕ16 mm × 81 mm (refer to Table 2 for mechanical attribute details). All electrical functional payloads and other onboard resources are housed inside the 3D printed biocompatible camera shell and are detailed in Section 4.

### 3.3. Stator Design and Fabrication

The stator generates controllable magnetic fields for camera actuation. Motor-driven permanent magnets have been adopted for this purpose over electromagnets which are bulky and prone to heat. As is schematically illustrated in Figure 3, the stator is equipped with three movable EPMs corresponding to the rotor IPMs. All EPMs pan together about $Z_{S}$ axis with respect to the stator housing. At the same time, the diametrically magnetized cEPM tilts about $X_{S}$ axis with respect to the axially magnetized end external permanent magnets (eEPMs). The two eEPMs are installed in an opposite manner to make their magnetic fields cancel out in the middle around the cEPM as indicated by the blue dashed lines. This configuration minimizes additional forces and torques exerted on the cEPM by eEPMs which will affect tilt motion of the cEPM.

**Figure 5:**
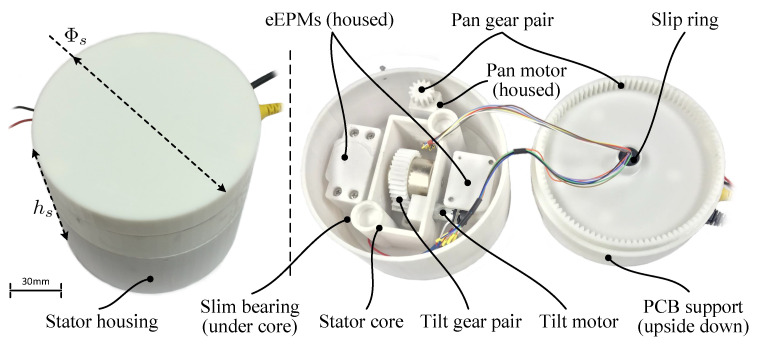
Mechanical design and fabrication of the stator: the assembled stator profile (**left**) and its inside mechanism (**right**).

Mechanical design of the stator is rapidly prototyped as shown in Figure 5. Overall profile of the stator assembly is a cylinder of ϕ120 mm × 108 mm. The stator core which carries all EPMs is fitted into the stator housing and seated on a thin slim angular contact ball bearing. Once installed, this bearing facilitates pan motion of all EPMs with respect to the stator housing. Meanwhile, the cEPM with a ring gear is installed on a shaft supported by the stator core and thus can rotate with respect to the eEPMs. Two DC servo motors are fixed on the stator core to, respectively, drive the pan and tilt rotations through gear pairs. After assembled, the printed circuit board (PCB) support will be bolted to the stator housing. Pan motion of the stator core is achieved through gear actuation between the pan motor gear and the inner gear in the PCB support while tilt driving is a straight-forward gear transmission from the tilt motor pinion gear to the cEPM ring gear. All electronic components which will be described in Section 4 sit on top of the PCB support and a 12-wire slip ring connector is utilized to prevent twisting of motor wires.

## 4. Tetherless Vision and Control

**Figure 6:**
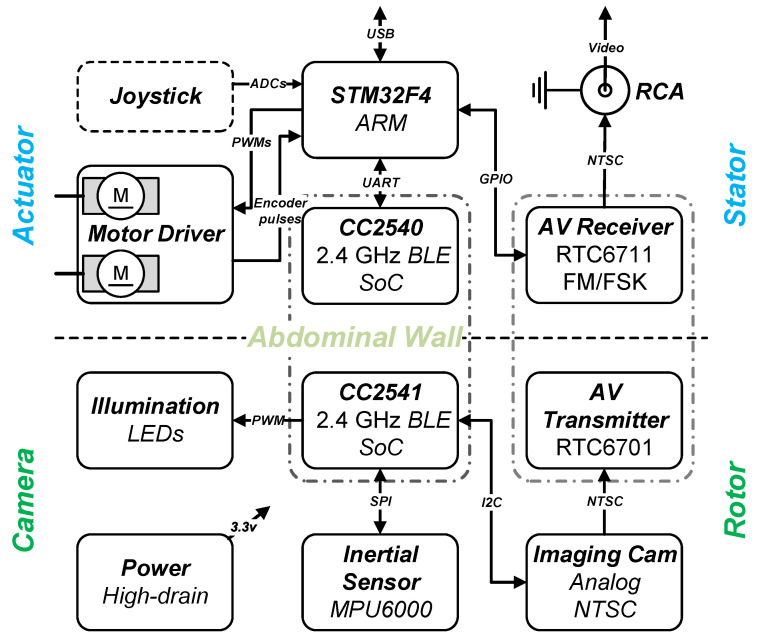
s-CAM electronic system architecture block diagram.

Tethering wires are usually necessary for video transmission, control communication, and power supply for insertable laparoscopic cameras. However, interference caused by these wires brings about operation restrictions and obstructs mobility of the camera. Furthermore, these wire bundles are difficult to sanitize and thus increase the chance of infection. Therefore, tetherless design and implementation carry practical meaning in making insertable laparoscopic cameras more clinically acceptable. This section elaborates the electronic solution which enables tetherless vision and control and contributes a fully functional s-CAM hardware architecture. As is shown in Figure 6, separated by the abdominal wall, the architecture is also divided into two parts: rotor camera and stator actuator. Although no physical connection exists in between, wireless control communication and video streaming have been established.

### 4.1. Rotor Camera

**Figure 7:**
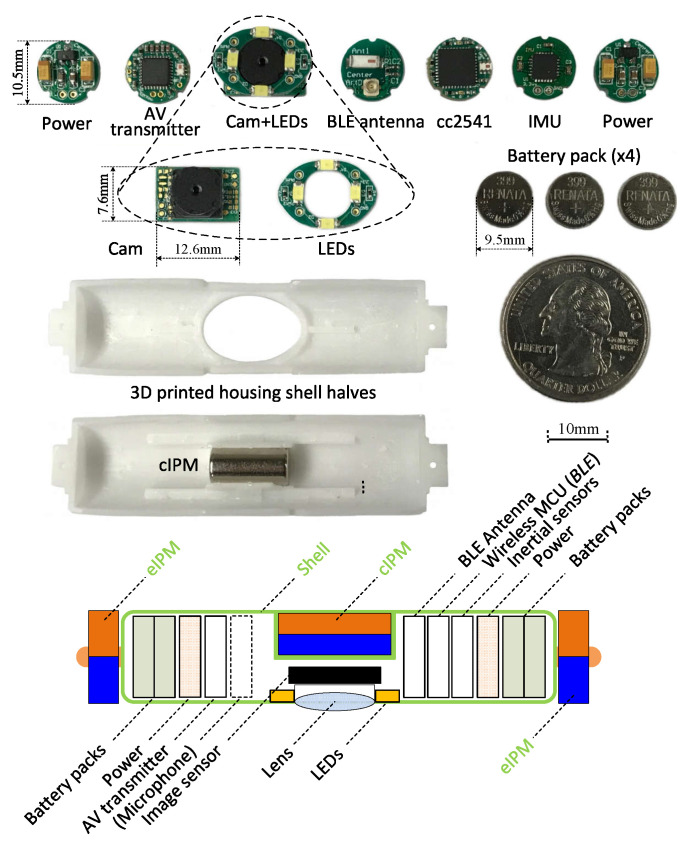
Implementation and layout of camera onboard modules.

As is interpreted in the lower part of Figure 6, electronic system of the rotor camera is built around a cc2541 wireless microcontroller unit (MCU), a low-power system-on-ship (SoC) solution for bluetooth low energy (BLE) applications. This cc2541 not only facilitates wireless stator-rotor communication in the ISM band for camera control but also governs all other resources onboard the rotor camera. Images captured by the imaging sensor are fed into an AV transmitter and then sent out over an embedded antenna. The cc2541 configures and tunes the imaging sensor online with I2C protocol for better imaging performance. Illuminating LED lights are controlled by PWM signals generated by an on-chip timer of the cc2541. Moreover, the cc2541 has access to an inertial measurement unit (IMU) through SPI for camera motion estimation. All the above components run on a 3.3v voltage regulated from onboard high-drain batteries. Design and implementation of the rotor camera electronic system represent a most challenging part of this research work. All onboard resources need to be sealed inside a stringently limited space in an extremely low profile. As is preliminarily unveiled in [41] and implemented in Figure 7, all camera payloads have been tailored into specific function modules. These modules are designed as round PCBs and stack up in a space-efficient manner inside the camera, except for the imaging sensor and the illumination LEDs which are facing sideways and fitted in under the cIPM. Fabricated function modules and onboard power batteries are presented in a disassembled view of in Figure 7. A microphone module is being integrated for audio feedback which, as reported [42], plays a very helpful role in improving surgical perception and operating confidence by allowing surgeons to hear the sounds of palpation, cutting, dissection, removal, as well as instrument vibration.

### 4.2. Stator Actuator

**Figure 8:**
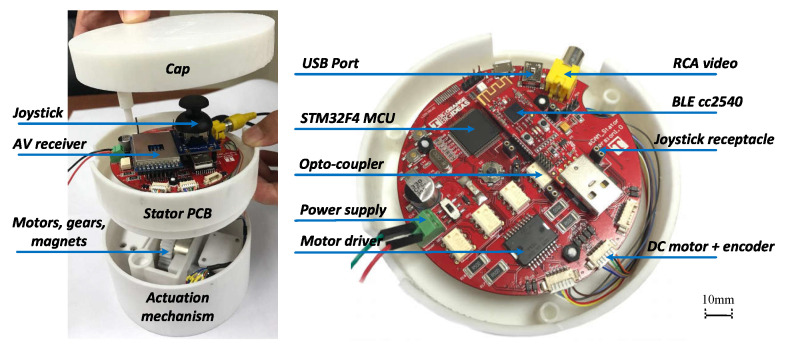
Implementation of the actuator electronic hardware.

As is illustrated in the upper part of Figure 6, electronic system of the stator actuator is centered at a 32 bit ARM Cortex-M4 microcontroller (STM32F4) . A cc2540 based BLE module is connected to the STM32F4 processor through UART to enable wireless stator-rotor control communication. The STM32F4 configures the AV receiver using GPIOs for different operation frequencies of wireless AV transmission. Video received by the AV receiver is accessed through RCA by the control unit and displayed for the surgeon’s reference. Facilitated by a dual full-bridge motor drive, encoders, and current sensing, closed-loop control of the pan and tilt motors is programed with the STM32F4 processor, which enables precise and safe camera actuation. A two-axis joystick can be plugged on and connected to the analog to digital converter (ADC) of the STM32F4 processor. Thus, manual control of camera pan and tilt motion is supported. In addition, a piezoelectric buzzer and several LEDs have been integrated to provide emergency alerts and warn surgeons of system status for safety concerns. Meanwhile, the actuator communicates with the control unit via USB. Figure 8 gives an explosive view of the actuator as well as a closeup of its electronic implementation. As is presented, the whole stator is designed as a two-story (ϕ120 mm × 108 mm, see Table 2) cylinder with a cap. The first story houses the actuation mechanism while the second story supports electronic hardware. The cc2540 BLE module is designed as a USB dongle with a postage package for multiple purposes. It can be either soldered on the stator PCB for stator-rotor communication or plugged onto a computer for BLE development and debugging. The stator actuator is powered with an external 12v DC power supply. Meanwhile, photocouplers have been employed between logic circuits and high-power circuits to improve system reliability. In case of control failure of the ECU, a joystick can be plugged into the receptacle to enable manual steering of the camera, which helps make this s-CAM fail-safe.

### 4.3. Modular and Reconfigurable Design

Hardware modularity and reconfigurability have been given special attention throughout design of the camera. Thus, these onboard camera modules can be easily interchanged for maintenance or reconfigured to build other devices dedicated for different purposes. For example, another rotor filled with batteries may be deployed alongside the camera to extend working time, or an illumination specific rotor equipped with only batteries and LEDs might be introduced for better surgical illumination, to name just a few. As a result, a family of these insertable devices may offer a systematical surgical solution in the future.

## 5. Reliable Embedded Software

System failure or malfunction occasionally occur even with the most advanced surgical robots such asthe *da Vinci* [43], which may cause adverse effects. No doubt reliability of software matters more for safety-critical medical applications than others. Thus, embedded software running on the s-CAM system should meet high safety and risk management requirements. While high-level planning and strategies run on the more powerful control unit, embedded software running on the stator and the rotor fulfills low-level tasks, communications, and control algorithms. Thus, embedded software must perform multiple s-CAM functions with a critical emphasis on software safety and reliability.

### 5.1. s-CAM BLE Application Profile

**Figure 9:**
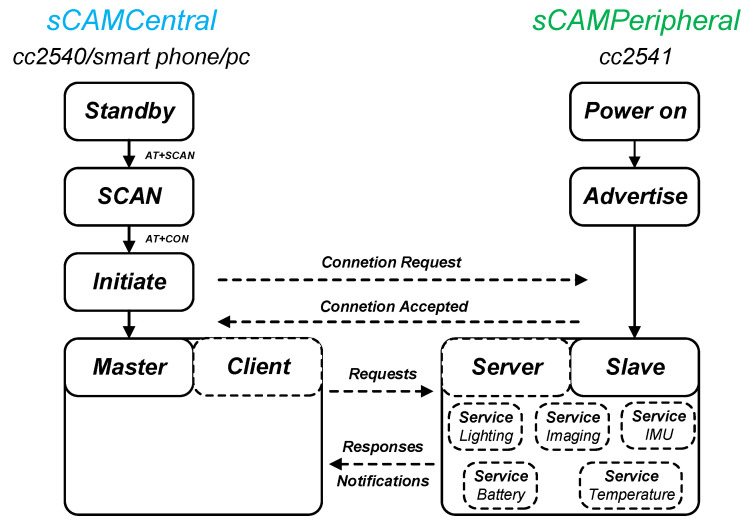
s-CAM BLE profile and application flow chart.

Bluetooth low energy (BLE, marketed as Bluetooth Smart) is part of the Bluetooth 4.0 standard targeting wireless healthcare and other applications with low-power, low-latency, and low-throughput features. Frequency hopping among 40 channels defined by the Bluetooth protocol counteracts RF interference and guarantees connection reliability. As a member of the Bluetooth Special Interest Group (BT-SIG), TI has designed and provided their BLE stack and cc254x series wireless SoCs for BLE user application development. Assisted by the TI BLE-Stack, a generic attribute profile (GATT) based proprietary s-CAM application profile has been developed for stator-rotor wireless communication and camera control. As is shown in Figure 9, a central-peripheral role configuration is adopted for stator-rotor BLE connection. The cc2540 onboard the stator is programmed as an sCAMCentral master while the cc2541 onboard the rotor works as an sCAMPeripheral slave. Once powered on, the peripheral device will periodically broadcast advertisements until a connection request is received from the central device. The central device is managed by the STM32F4 through UART using AT commands as is illustrated in Figure 6 and Figure 9. The sCAMCentral will initiate a connection request to the sCAMPeripheral when the peripheral device is found. If the request is successfully accepted, connection between sCAMCentral and sCAMPeripheral will be established after a mutual parameter update. Once connected, the master works as a data client while the slave works as a data server. The sCAMPeripheral provides services related to camera onboard resources including lighting, imaging, IMU, temperature and battery. The sCAMCentral requests these services so as to realize wireless control of the camera. More meaningfully, more than one sCAMPeripheral slave may be connected to the sCAMCertral master to form a star topology multi-camera network, drastically augmenting the system capabilities.

### 5.2. Real-Time Software Framework

An embedded real-time operating system (RTOS) is necessary for the following reasons. First, most low-level control and processing algorithms are being executed on the STM32F4 ARM processor, which is too complex to be implemented just in one simple programming loop. Second, processing time matters particularly for this safety-critical application and failure of timely event response may cause serious medical disasters. Third, application programming and debugging on a bare MCU is time consuming, error prone, and code inefficient. An RTOS supports multitasking, is time sensitive, and bounds event responses within fixed time constraints. Meanwhile, the operating system encapsulates hardware resources into system services and APIs to expedite software programming.

**Figure 10:**
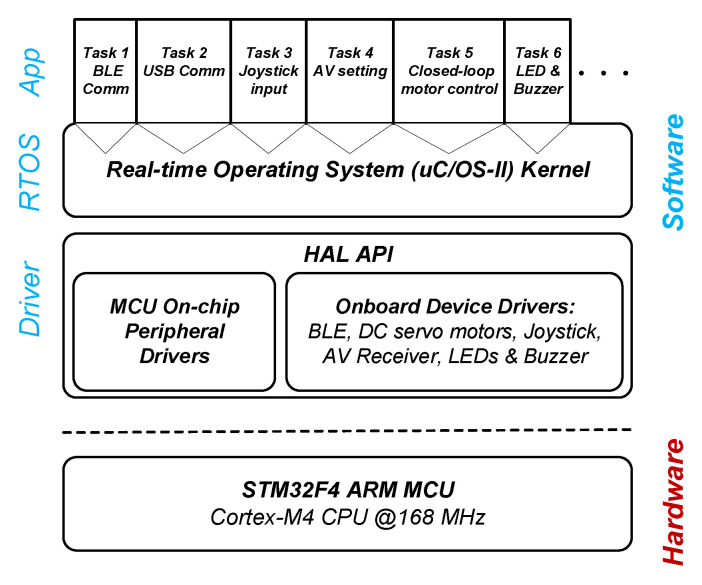
Real-time operating system based software framework.

μC/OS from Micrium^®^(acquired by Silicon Labs^®^ in 2016) is a lightweight scalable embedded RTOS which features industrial level reliability and application efficiency. Most importantly, μC/OS has been verified on many hospital devices used solely by medical professionals, which all have met medical software safety certification standard (IEC 62304) and received FDA 510(k) clearance. The event based preemptive multitasking kernel of μC/OS schedules up to 256 tasks in real-time according to their statuses and priorities. As is shown is Figure 10, on top of the hardware abstract layer (HAL), the RTOS manages all on-chip and onboard resources. Facilitated by the multitasking kernel, different functions of the s-CAM have been designed as individual tasks sharing the MCU resources and more tasks can be added easily in future when necessary. Currently, six tasks have been developed to fulfill all s-CAM functions: BLE communication task, USB communication task, joystick input processing task, AV receiver configuration task, closed-loop DC motor control task as well as LED and Buzzer task. Each task was assigned a different priority and the RTOS kernel guarantees that the ready task with the highest priority always gets executed. By delicate priority assignment and task loop design, all tasks run on the RTOS reliably and efficiently as if every task has its own CPU. Above all, real-time event response and processing within a fixed time have guaranteed reliability and safety of the s-CAM.

## 6. Experiments

**Figure 11:**
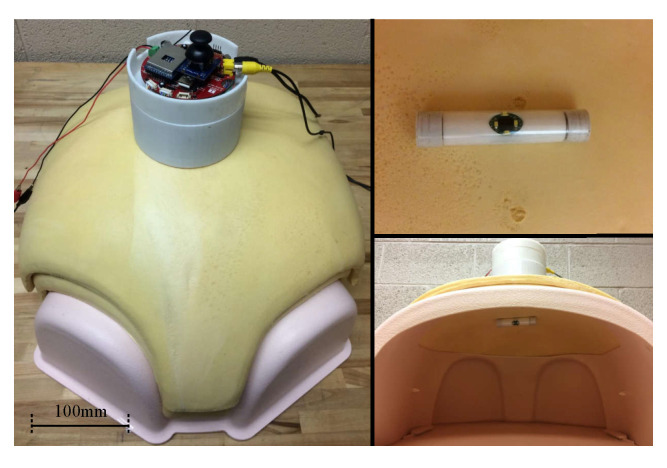
Ex vivo phantom experiment setup in a 3-Dmed^®^ synthetic abdomen model.

Experimental tests of s-CAM functions have been performed ex vivo using a 3-Dmed^®^ phantom human abdomen in order to evaluate the s-CAM performance and verify design feasibility. The phantom abdomen has soft covering material simulating physical properties of abdominal wall to practice trocar placement and insertion. It also has a slide-out drawer allowing easy access to the cavity. Wall thickness of the abdomen model was set to about 30mm which, according to studies [40,44], represents an average value of human abdominal wall thickness. As is shown in Figure 11, the synthetic abdomen model was laid on a horizontal workbench while the stator was placed right over the abdomen and the rotor camera was introduced into the abdominal cavity through the drawer opening on the model. Magnetic coupling between the camera and the actuator was established under direct visual assistance and the camera was anchored against the interior abdominal wall. Vision and control of the camera were using wireless communications between the stator and the rotor. The stator ran on a 12v DC power supply and communicated with the ECU (a windows PC) through a USB cable. Meanwhile, video signals were output to the ECU using a coaxial composite video cable.

### 6.1. Tetherless Laparoscopic Vision

**Figure 12:**
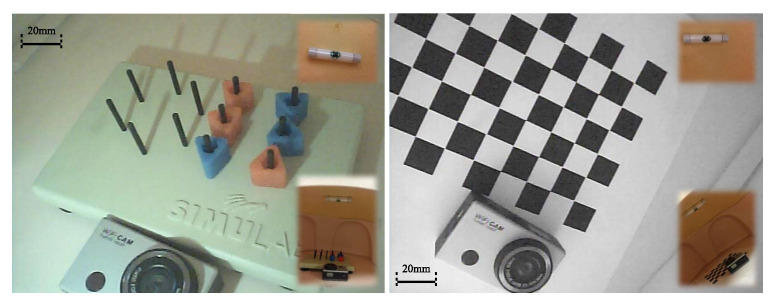
Wireless imaging performance test. A picture of the anchored s-CAM taken by a wifi camera is shown in a top-right insets. (**Left**: Color imaging of a Peg Transfer Board; **Right**: Monochrome grid imaging).

Wireless video received by the AV receiver was fed into a video to usb converter (DFG/USB2pro) which connects to the ECU. Video streams was displayed, processed, and stored using the monitoring software and development APIs provided with the converter. As is shown in Figure 12, two experiments have been performed in order to evaluate the wireless imaging performance. First, a SIMULAB Peg Transfer Board with colored triangles was placed in the phantom model for evaluation of color imaging quality. Then, a mocochrome grid was used to check image distortion of the camera. An LED light meter (Extech LT40) was employed to measure the environment illumination level in the abdominal cavity and a Wi-Fi camera was placed inside to capture motion of the camera.

Results suggested that both color and monochrome objects were agreeably imaged for human eyes under appropriate illumination over 500 lx. Color images became monochrome when the illumination fell below 200 lx. No noticeable image distortion was observed by human eyes and wireless video connection was stable throughout the test of 30 min in the lab. Meanwhile, we deliberately separated the AV receiver from the camera up to a distance of 10 m, which gives some idea of the maximum signal coverage. A lens cleaning and debris prevention solution was also shared in our previous work [45] to prevent the camera from getting blurred by peritoneal fluid.

### 6.2. Tetherless Camera Control

Tetherless camera control based on BLE communication was evaluated in terms of s-CAM profile services and the received signal strength. As is detailed in Section 5.1, camera functions are all implemented as BLE profile services including lighting, imaging, IMU, battery, and temperature. Each service has its own characteristic data bytes that can be read or written by the BLE central device on the stator. Results had confirmed effective control of camera functions using these BLE services. Illumination LED PWM was set arbitrarily between 0 (off) and 255 (fully lit) by writing the lighting service byte. Imaging quality could be tuned online by writing the imaging service bytes, which actually updates the CMOS imaging sensor registers. By reading the IMU service bytes, motion information of the rotor was acquired in real time at 30 frames per second, which was able to feedback close-loop camera motion control. Likewise, battery voltage and temperature of the camera were monitored by reading corresponding service bytes, respectively.

**Figure 13:**
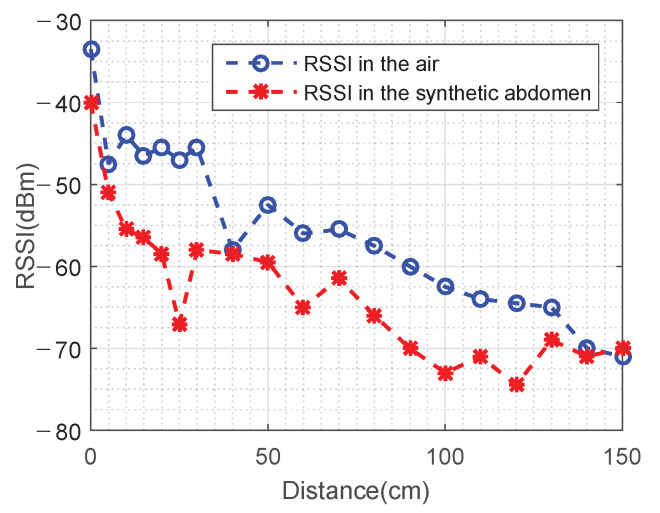
RSSIs with respect to stator-rotor distances.

Recieved signal strength indicator (RSSI) values on the BLE central device were recorded and graphed as Figure 13. First, both the actuator and the camera were placed in the open air. Then, the camera was inserted into the abdominal cavity while the actuator was left in the outside. RSSI values at different stator-rotor distances in both scenarios were compared to show radiation property of BLE signals and affects of the abdomen model. It was concluded that RSSI was attenuated to a certain degree by the abdominal model material. Noticeable differences were seen primarily in the middle range from 10 cm to 140 cm while RSSI values were similar between these two scenarios in ranges within 10 cm or beyond 140 cm. Since the camera is actually anchored close to the actuator in practice so as to maintain effective magnetic coupling, an RSSI value of −52 dBm or better will be achieved within 50 mm.

### 6.3. RTOS Based Software

Six user tasks were running on the embedded real-time operating system as developed in Section 5.2. Each task was programmed as an infinite loop with a unique priority and delay time, which were assigned as Table 3. The smaller the number, the higher the task priority. Each task delays itself an appropriate time periodically so that tasks with lower priorities also get executed. System tick clock was set at 1KHz for the RTOS and task event response within 1ms was ensured for the ready task with the highest priority. USB communication with the ECU was using the interrupt service. In this way, commands from the control unit are processed in microseconds. CPU usage was about 3∼5% when all tasks were in full operation, which indicates a great processing potential for more tasks.

### 6.4. Smart Illumination

**Figure 14:**
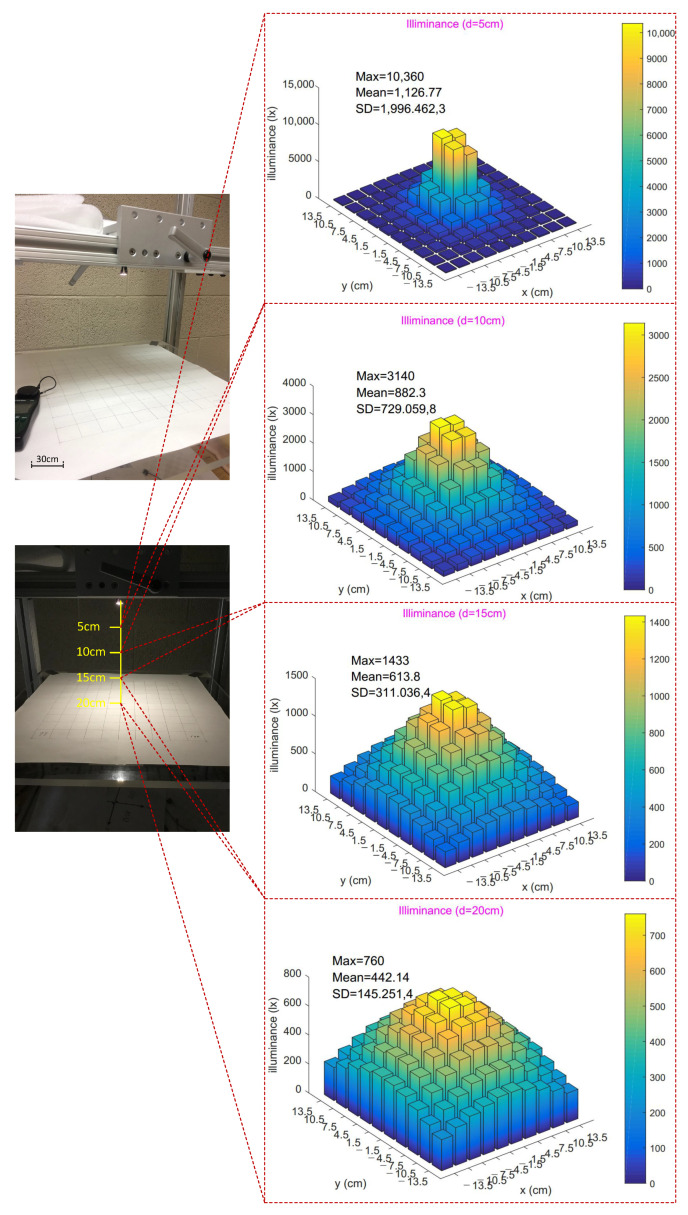
Illuminance tests at different distances to the LED module.

The laparoscopic camera is usually hovering around 12 cm∼15 cm above a surgical area of no largers than 15 cm × 15 cm in clinical practice [46,47]. Illumination tests were performed using an adjustable frame built with the t-slotted 80/20 aluminum structural material as shown in Figure 14. The LED illumination module was hovering right above a 30 cm × 30 cm square plane which was divided into 100 small squares of 3 cm × 3 cm. The distance between the illumination module and the square plane was adjusted to 5 cm, 10 cm, 15 cm, and 20 cm, respectively, for 4 tests. Each test was repeated 3 times and averaged illumination level of each small square was recorded using the LED light meter (Extech LT40, FLIR Commercial Systems Inc., Nashhua, NH, USA). All tests were carried out in a natural dark environment and the LEDs were fully lit with a PWM of 255.

Experimental results were visualized using 3D bar graphs in Figure 14, which unveiled radiation properties of the LED illumination module. High illumination levels were all in the center of the plane for each test with a highest of 10,360 lx seen in the 5 cm test. As the testing plane moved away from the LED module, illumination levels decreased and light flux became more evenly distributed. The lowest mean illumination level was 442.14 lx with the 20 cm test, which still facilitates acceptable imaging performance according to the tetherless laparoscopic vision test in Section 6.1. This exceptional low-light performance should be attributed to the high sensitivity of the imaging sensor. Average illumination within a 15 cm × 15 cm plane at a distance of 15 cm was above 963 lx, which is sufficient for effective imaging according to the experiment results. An algorithm adjusts the PWM value to adjust the lighting level of LEDs in a smart way to ensure agreeable imaging at lowest possible power consumption.

### 6.5. Power Duration

Another characteristic of interest is power duration, which determines how long the camera can continuously function and thus the scope of procedures that may be performed using this camera. Power consumption of each module onboard the camera has been tested and tabulated in Table 4. Average power duration may reach more than 50 min without any power optimization. This power duration time covers most diagnostic laparoscopic procedures and some simple operative laparoscopic procedures [48]. In the future, wireless charging or powering might be an option to extend battery duration or eventually eliminate the power limit.

### 6.6. Camera Mobility

**Figure 15:**
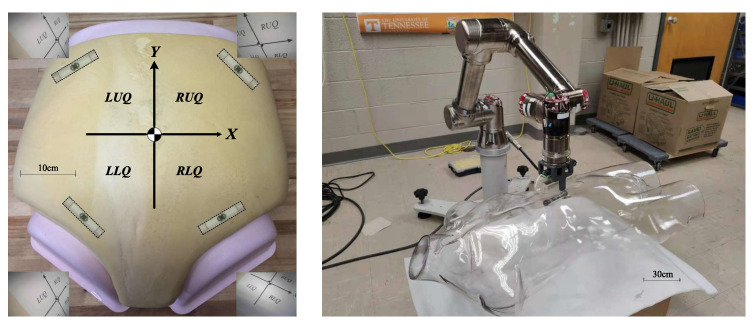
Translation of the camera and another setup for mobility test. A multi-quadrant coordinate frame was placed in the belly for imaging reference. Left upper quadrant (LUQ), left lower quadrant (LLQ), right upper quadrant (RUQ), right lower quadrant (RLQ).

Multi-DoF camera mobility was tested using the same setup as Figure 11 and also another setup with a robot arm using a transparent abdomen as shown in Figure 15. Translation of the camera was tested by moving the actuator over the surface of the abdomen models while pan and tilt of the camera were tested by driving the external magnets. The IMU data onbaord the s-CAM was able to shed some light on quantitative motion information.

**Figure 16:**
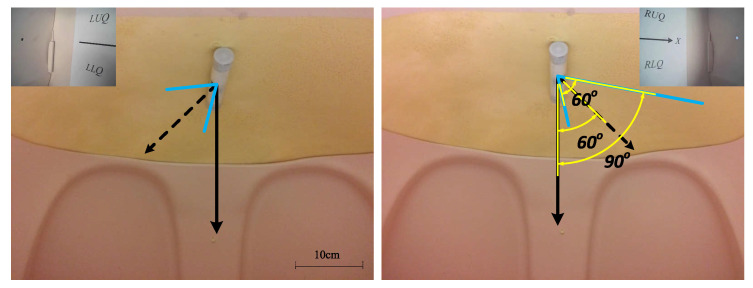
Tilt motion of the camera. A resultant tilt observation range angle of ±90${}^{\circ }$ was achieved.

Thanks to the non-contact actuation and teteherless control, dexterous camera mobility was observed in the test results. Translation of the camera could reach multiple quadrants inside the abdomen model as shown in Figure 15. Velocity of translation movement was about 1 cm/s at maximum. Pan motion range of the camera was 0∼360${}^{\circ }$. Results also confirmed that the camera could tilt between −180${}^{\circ }$ and +180${}^{\circ }$. However, it’s worth noting that the camera only needs to tilt between −60${}^{\circ }$ and +60${}^{\circ }$ in clinical practice. As is illustrated in Figure 16, the camera has a 60${}^{\circ }$ field of view which provided a view scope of ±90${}^{\circ }$ aided by a ±60${}^{\circ }$ tilt angle, sufficient for the camera to observe the whole abdominal cavity in the tilt DoF. A 15${}^{\circ }$ lag between the actuation and rotor was observed during pan motion and the pan speed may reach up to around 30${}^{\circ }$/s. Tilt motion was smoother than pan and showed a faster maximum of around 60${}^{\circ }$/s.

## 7. Conclusions and Future Work

A novel insertable laparoscopic camera characterized by tetherless vision and control with non-contact actuation has been introduced, implemented, and verified in this paper. Wireless vision with exceptional low-light performance has been achieved in the NTSC format and camera control is also realized wirelessly through BLE communication. Consequently, cumbersome tethering wires have been completely eliminated for this insertable camera and flexible camera mobility have been demonstrated. Modular and reconfigurable design of camera function modules provides a hardware reference for development of more potential insertable medical devices. Embedded software design based on the certified TI BLE-Stack and the well-approved μC/OS RTOS guarantees reliability of the safety-critical medical device. Camera functions have been designed as services of a proprietary BLE profile for the s-CAM camera and stable BLE connection for camera control is confirmed. The RTOS based software framework reliably responds to task events within fixed time constraints, which helps avoid adverse effects caused by unpredictable responding times. We also have improved the non-contact camera actuation design from our previous work by using pure permanent magnets. Thus, multi-DoF decoupled camera mobility has been realized in a simpler and more compact manner.

At present, the s-CAM is a proof-of-concept prototype whose technical feasibility and basic functions have been experimentally verified ex vivo in our lab. More meaningful work needs to be done in the future to further evaluate efficacy of the system and bring it closer to clinical applications. First, both surgical and training task-based experiments will be performed using the s-CAM to assess its efficiency in practice. Second, pose estimation of the insertable camera will be realized to support closed-loop precise camera motion control, which will significantly augment laparoscopic vision. Third, interaction force between the camera and the abdominal wall tissue needs to be measured so that an appropriate camera–tissue contact force can be maintained and the camera will neither fall off nor damage the tissue.

## Figures and Tables

**Table 1:** Physical attributes of magnets ${}^{1}$.

Magnets	Outer Diameter	Inner Diameter	Hight	Mass	Grade
eEPM(×2)	25.4 mm	N/A	25.4 mm	96.5 g	N52
cEPM	25.4 mm	6.35 mm	25.4 mm	90.5 g	N42
eIPM(×2)	12.7 mm	4.76 mm	6.35 mm	5.19 g	N42
cIPM	6.35 mm	N/A	12.7 mm	3.02 g	N42

^1^ All are NdFeB based permanent magnets.

**Table 2:** Physical attributes of the stator and the rotor.

Symbol	Description	Value
$\Phi_{s}$	Stator diameter	120 mm
$h_{s}$	Stator height	108 mm
$m_{s}$	Mass of stator	762.3 g
$\Phi_{r}$	Rotor/tube diameter	16 mm ${}^{1}$
$l_{r}$	Rotor length	81 mm
$\phi_{c}$	Camera body diameter	12.5 mm
$h_{c}$	Camera body length	68 mm
*a*	Window length	16 mm
*b*	Window width	10.8 mm
$m_{r}$	Mass of rotor	37.5 g
$l_{1}$	e(x)PM distance	72.5 mm
$l_{2}$	cIPM offset	4.35 mm
*d*	Stator-rotor distance	variable ${}^{2}$

^1^$\Phi_{r}$ is being further reduced to around 10.5 mm. ^2^ Affected by the abdominal wall thickness [40].

**Table 3:** Task priorities and delay times.

Tasks	Priority	Delay Time (ms)
BLE Comm	4	50
USB Comm (Int.)	5	50
Joystick Input	6	50
AV Setting	7	50
Motor control	8	20
LED and Buzzer	9	50

**Table 4:** Power consumption ratings.

Module	Tested Current (mA)	Max Current (mA)
Wireless MCU	17.5 (Active TX)	18.2 (Active TX)
Inertial Sensors	2.9	3.9
Imageing	55	60
AV Transmitter	50	54
Illumination	0∼120	130

## Data Availability

Not applicable.

## Abbreviations

The following abbreviations are used in this manuscript:

s-CAM	Surgical camera
BLE	Bluetooth low energy
RTOS	Real-time operating system
LS	Laparoscopic surgery
SILS	Single incision laparoscopic surgery
MLS	Multi-port laparoscopic surgery
ECU	External control unit
CVS	Critical view of safety
MIS	Minimally invasive surgery
EPMs	External permanent magnets
IPMs	Internal permanent magnets
cIPM	Central internal permanent magnet
eIPM	End internal permanent magnet
cEPM	Central external permanent magnet
eEPM	End external permanent magnet
PCB	Printed circuit board
MCU	Micro-controller unit
SoC	System-on-chip
IMU	Inertial measurement unit
